# Evaluating quality of life in families with Williams Syndrome patients

**DOI:** 10.1186/s12955-021-01704-0

**Published:** 2021-04-14

**Authors:** Esther Moraleda Sepúlveda, Patricia López Resa

**Affiliations:** Health Sciences Faculty, Castilla-La Mancha University (Spain), Avda Real Fábrica de la Seda s/n. 45600 Talavera de la Reina, Toledo, Spain

**Keywords:** Williams Syndrome, Family, Quality of life, Evaluation

## Abstract

**Background:**

Williams Syndrome is a developmental disorder characterized by a variable intellectual disability. People with Williams Syndrome need the intervention of several clinical and educational specialists throughout their life. However, little is known about the impact produced by this disability in their immediate environment, especially in families. The purpose was to know the level of quality of life described by families with Williams Syndrome.

**Methods:**

The sample was made up of 33 families belong to Spanish Williams Syndrome Association who were evaluated using the Kidslife Scale. Their children and adolescents were between 4 and 20 years old. Eight main quality of life domains were evaluated: emotional well-being, physical well-being, material well-being, personal development, interpersonal relations, social inclusion, self-determination and rights

**Results:**

The obtained data indicated that the degree and presence of intellectual disability did not homogeneously influence people’s quality of life, but many variables could alter their quality of life to a greater or lesser extent. There are no significant differences between quality of life areas but significant differences appeared for level of dependence in the self-determination subarea (*p* < .05).

**Conclusions:**

These results led us to analyse the social and emotional implications for families and their environment.

## Introduction

Williams Syndrome (WS) is a neurodevelopment disorder that occurs in one in every 7500 live newborns [1], brought about by the de novo deletion of 26–28 genes from chromosome 7 q11.23 [2]. Clinically speaking, WS is characterised by some typical facial features, and by slight, or moderate and asymmetric, intellectual disability (ID), with marked deficits in certain areas (psychomotricity, visuo-spatial integration, reduced attention capacity, concentration) and they relatively preserve others (language and musicality), friendly personality, occasional hypercalcaemia in infancy and vasculopathy with supravalvular aortic stenosis [3]. Their ID means their average Intelligence Quotient (IQ) is 55–60 points, but may vary between 40 and 100 points [4]. This very wide variability means that any administered therapies must be carefully and individually adapted to each person with WS [5].

Regarding conduct characteristics, people with WS tend to be associated with a high degree of anxiety, specific phobias and attention deficit hyperactivity disorder (ADHD), which can negatively affect their quality of life (QoL), particularly in adults [6]. Parents perceive them as being active, in good spirits, well-balanced, shy, over-friendly and vigorous. No gender or age differences have been found for them. All these prototype clinical characteristics of WS may have implications for WS patients’ day-to-day lives, and also for their environment, which influences their QoL [7].

The term QoL refers to a concept that may significantly vary in perception terms depending on the period, social group and culture that the individual belongs to [8, 9]. Nonetheless, most approaches have demonstrated the importance of corroborating the biopsychosocial nature of human beings and evidenced the integrating nature of this concept [10]. In line with this, Schalock and Verdugo [11, 12] have proposed an influential and generalisable QoL model which, when applied to people with ID, attempts to positively impact many factors of their life [13]. Likewise, several researchers have considered the possible impact that having ID has on people’s environment based on the aforementioned biopsychosocial conception [14, 15]. Accordingly, Gràcia and Vilaseca [16] stated that families point out many weak points in their QoL as to the possibility of relying on counselling, specialists or parent groups with which they can plan suitable activities for their children. So it would appear that the family organization, their beliefs, and the context they live in, are variable that affect the amount of supports families need [17].

The family QoL concept has emerged considerably in the last decade as a decisive construct to know the impact of having a family member with a disability, and to evaluate the possible outcome of the services and support that families receive [18, 19]. In addition, most research focuses on the family OoL, but not so much, on that of the person with a disability themselves. However, it seems clear that QoL of person with a ID has a direct influence on family QoL [20]. Nevertheless, research to date on the QoL of families with sons or daughters with WS is virtually inexistent despite the importance that knowledge on this aspect might have on families’ QoL. Therefore, a consensus has been reached by the scientific community on the desire of families with relations affected by WS to not talk so much about what these patients are unable to do, but about what they could achieve with suitable support [21].

## Methods

### Participants

Thirty-three parents with children diagnosed with WS aged 4–20 years participated in this study, and they belonged to the Asociación Williams España (ASWE; Spanish Williams Association). They included 26 mothers and 7 fathers. The age of the parents was between 30 and 60 years. 23 of them are families with children between 4 and 12 y 10 are families with adolescents between 13 and 20 years old.

### Instrument

The KidsLife Scale evaluates the QoL of children and adolescents with ID [22]. It evaluates personal outcomes related to QoL in children/adolescents with ID aged between 4 and 21 years. It is a set of questions about observable QoL aspects that can be answered by an external observer who knows the child well and has the chance to observe him/her for long periods of time in different contexts. The scale comprises 96 items and provides standard and percentile scores for eight main QoL domains: emotional well-being; physical well-being; material well-being; personal development; interpersonal relations; social inclusion; self-determination; rights. Items were selected from the studies by Petry et al. [23, 24] and from the San Martín Scale [24]. Each item presents four options and only one must be chosen (never, sometimes, frequently or always). Evidence of convergent validity is given by the magnitude, directionality and statistical significance of the standardized coefficients (in all cases they are higher than 0.70, positive and significant with *p* < 0.01). Regarding the reliability of the scale, in Cronbach’s alpha coefficients, the total scale obtained a coefficient of 0.96, while the domains ranged between 0.80 (physical well-being) and 0.90 (personal development).

The results were analysed in accordance with three variables included in the KidsLife scale: level of dependence (moderate, severe and high); level of need (limited, intermittent, extensive and generalised); level of disability (mild, moderate, severe and profound). The data on these three variables are answered by family members in the first part of the questionnaire and correspond to the information on personal characteristics.

### Procedure

The ASWE was firstly contacted to inform it about the project and to know if it might be interested in participating. When the ASWE agreed, it was sent informed consent for the mothers and fathers of offspring with WS belonging to the ASWE. Informed consent was approved by the corresponding university’s Ethics Committee. After signing this document, they were sent the written questionnaire that they had to complete. All the participants filled in this questionnaire individually during a single session lasting 20–30 min.

The normality of the sample was verified through the Kolmogorov–Smirnoff test, resulting in a non-parametric test. The results were then analyzed using the U-Mann Whitney test. To analyze the correlation between variables, Spearman’s Rho test was used.

## Results

The data didn't show significant differences in standard scores in the various subareas making up the scale (*p* > 0.05): social inclusion (X = 34.02, SD = 5.79); self-determination (X = 31.76, SD = 5.98); emotional well-being (X = 37.71, SD = 6.28); physical well-being (X = 38.8, SD = 4.55); material well-being (X = 42.21, SD = 4.53); rights (X = 39.97, SD = 6.14); personal development (X = 38.65, SD = 6.22); interpersonal relations (X = 36.47, SD = 6.43). In Table 1, the results can be seen depending on the variables.

**Table 1:** Medium scores in QoL domains

	Social inclusión	Self-determination	Emotional well-being	Physical well-being	Material well-being	Rights	Personal development	Interpersonal relations
*Level of dependence*								
1	35.45(5.3)	31.64(5.5)*	37.36(4.2)	38.72(4.5)	42.81(4.2)	37.18(9.8)	35.63(8.3)	34.36(7.6)
2	33.71(4.7)	30.85(6.8)	37.64(5.1)	39.57(5.1)	42.28(5.2)	40.64(5.6)	38.92(6.8)	37.21(6.5)
3	36.2(4.3)	29.40(2.9)*	40.80(7.1)	41.40(5.9)	42.40(4.1)	40.40(5.7)	41.40(5.0)	37.40(7.2)
*Level of need*								
1	38.5(4.9)	33.50(9.2)	42.50(2.1)	40.50(0.7)	43.50(4.9)	47.00(1.4)	42.00(8.5)	36.00(5.6)
2	29.66(5.8)	27.66(6.2)	32.00(5.7)	33.86(5.1)	36.66(5.5)	35.00(6.8)	33.33(4.5)	32.80(4.3)
3	64.66(5.9)	29.00(6.3)	37.16(4.8)	39.16(2.6)	42.66(3.8)	36.83(10.9)	35.16(9.2)	32.83(9.1)
4	34.37(5.4)	29.50(4.6)	42.75(6.8)	39.50(6.8)	41.75(4.7)	38.37(5.8)	40.00(6.9)	31.12(6.4)
*Level of disability*								
1	37.25(5.4)	33.16(6.2)	38.50(5.1)	44.08(3.5)	43.08(4.4)	10.36(9.0)	13.19(8.5)	12.81(7.8)
2	33.41(5.0)	30.50(4.8)	37.33(4.9)	37.25(5.0)	41.16(5.5)	39.00(6.9)	38.58(5.7)	37.00(5.0)
3	32.50(5.3)	27.16(5.0)	38.66(8.4)	41.00(6.4)	44.00(3.0)	39.16(6.5)	40.50(5.3)	38.66(6.2)
4	0	0	0	0	0	0	0	0

Standard deviations are given in parentheses

Level of dependence (1 = Moderate; 2 = Severe; 3 = High)

Level of need (1 = Limited; 2 = Intermittent; 3 = Extensive; 4 = Generalized)

Level of disability (1 = Slight; 2 = Moderate; 3 = Severe; 4 = Extreme)

*Significant differences *p* < .05

The results indicated that significant differences appeared for level of dependence in the self-determination subarea with the parents whose son/daughter had WS and a moderate degree of dependence compared to those with a child with WS presenting a high level of dependence (*p* < 0.05).

The results were also analysed according to subjects’ ages, who were distributed into two age groups: 4–12 years and 13–20 years. No differences were found between the two age groups for all the evaluated domains.

## Discussion

The present study aimed to analyse the QoL of people with WS from the point of view of families. This data obtaining a QoL profile allows support to be individualised planned [25]. To do so, we started by taking the most recent conception of disability, understood as the result of the interaction between someone and their environment insofar as the availability of support would significantly favour their level of functioning [26, 27]. The obtained data were very interesting because they informed that it was not the degree of disability that determined the family’s QoL, rather the presence of disability itself. The same can be stated for the degree of dependence and the degree of need. The presence of WS predisposes families to a specific profile, without taking into account other variables such as the degree of disability, dependence or need. Families do not perceive their QoL as being different according to these variables, which indicates a more homogeneous profile for the implications involved in being a family with WS. It is also worth stressing that parents’ perception does not change about their child’s evolutionary development, rather a certain consensus exists about the most outlined difficulties according to the results found.

Regarding the QoL domains the results obtained about the social inclusion domain revealed that, despite more visibility, this groups’ social inclusion remains a pending issue, which agrees with the publications by Escudero and Martínez [28] and by Gómez et al. [29].

Next comes what could be the main limitation for QoL according to the obtained data: mastering self-determination [30] although there are no significant differences. Wehmeyer and Garner [31] stated that important decision making, like education centres or type of schooling, is made unilaterally by parents in infancy and adolescence, and the person with ID is not involved. Thus our data demonstrate that some differences exist for level of determination according to degree of dependence. Likewise, the results provided by families do not agree with those reported in other research works, which reflect that decision making is significantly linked with degree of disability [32]. Therefore, the degree of disability does not seem to influence the family. This could be due to the fact that they do not give as much importance to the individual differences that disability can bring. Families consider in a global way that person with WS is a person with disability but it isn´t important degree of disability.

The results of the emotional well-being, physical well-being and material well-being domains indicate a moderate degree of satisfaction in these subareas [33, 34]. These results do not coincide with other research works, which state that the conduct problems stemming from emotional upsets tend to negatively impact both family and individual well-being [35]. Also in relation to these categories, Guyard et al. [36] stress the increased economic expense of families with relations with ID, which negatively influences family QoL. Therefore, the results found in our research are different from those found by other authors. This could be due to cultural and socioeconomic differences that have not been controlled.

Once again the results given by parents for the rights dimension do not coincide with studies that have related this subarea to degree of disability. Turnbull and Turnbull [37] add that, when disability is slight or moderate, parents tend to point out that people with ID have very few opportunities and that support is lacking to allow them to exercise their rights. Thus to a certain extent, families still perceive their children as being defenceless, which might sometimes lead to overprotection [38].

Regarding these individuals’ personal development, the obtained data stress that parents generally assume that their offspring follow a continuous learning process of social skills, which are essential for personal development and, therefore, for a more than satisfactory QoL [39].

The interpersonal relations dimension results tends to be worse than others domains because, in general, both people with ID and their families have fewer relations, and are more prone to the social isolation risk [40].

It is also worth stressing that most of the scales were filled in by mothers, as opposed to a small percentage of fathers. Most of QoL surveys are in general designed to be administred by the “main caregiver”. This indicates that mothers are the main “carer” or reference person. In line with this, Antó et al. [41] state that the care responsibility is shouldered by mothers as they are in charge of their day-to-day lives. Rentería et al. [42] add that mothers are perceived to play a role that provides affection and care and, therefore, family stability. Family quality of life is a dynamic and relational concept and the way in which individual members work can influence the overall well-being of the family [43]. This can be due to sample size.

Families perceive that the QoL of individuals with ID may differ from that held by professionals close to these patients [44]. Hence it would be interesting to complement our data with those provided by professionals working with people with WS on a daily basis.

## Conclusions

To conclude, it is difficult to homogenise everyone with ID (in our case WS) and their families in the QoL concept because this is strongly influenced by the several factors that shape personal well-being [12, 45, 46]. Nonetheless, it is very important to consider these aspects when offering the necessary support and resources to help families throughout the lifetime of people with WS.

## Data Availability

The datasets used and/or analysed during the current study are available from the corresponding author on reasonable request.

## Abbreviations

QoL: Quality of life
WS: Williams Syndrome
